# Supplementary material for: GIANI – open-source software for automated analysis of 3D microscopy images
Source: J Cell Sci. 2022 May 17;135(10):jcs259511. doi: 10.1242/jcs.259511 (PMC9189431; doi:10.1242/jcs.259511)
Supplement: Supplementary information [file joces-135-259511-s1.pdf]

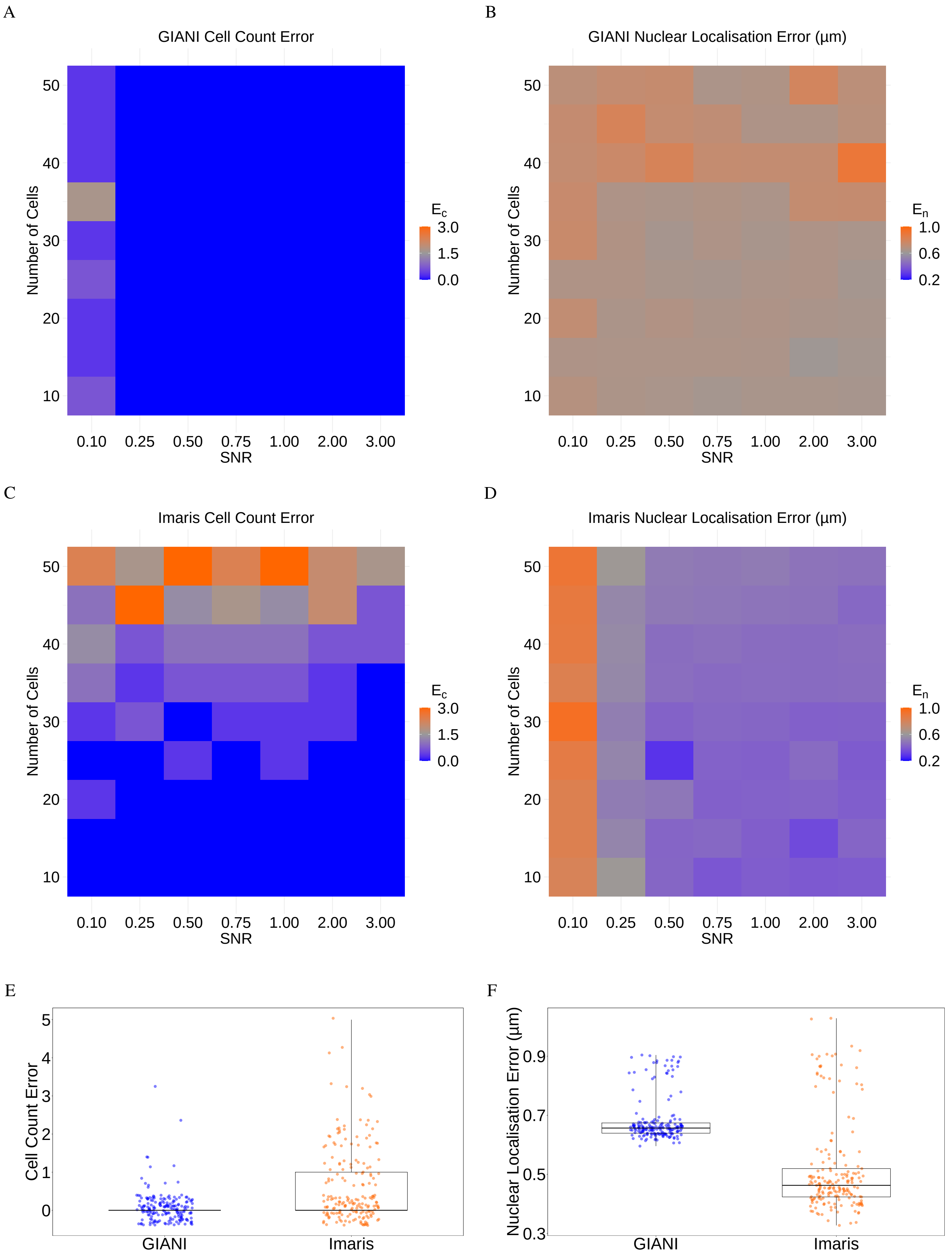

Journal of Cell Science • Supplementary information

Fig S1. **Accuracy of simulated nuclei detection and localisation by GIANI and Imaris.** In each of the heat maps, a single tile represents the average of three simulated embryos. The GIANI and Imaris settings used to produce this data are available to download from https://doi.org/10.5281/zenodo.6206087. A: Absolute errors in cell counts (E_c) produced by GIANI for simulated embryos with the indicated number of cells and signal-to-noise ratios (SNR). B: Absolute errors in nuclear centroid localisation (E_{nl}) produced by GIANI. C: Absolute errors in cell counts produced by Imaris. D: Absolute errors in nuclear centroid localisation produced by Imaris. E - F: Comparison of the overall distribution of errors in cell counts and nuclear centroid localisation GIANI and Imaris, across all SNRs and cell numbers. Each dot represents a single simulated embryo.

Journal of Cell Science • Supplementary information

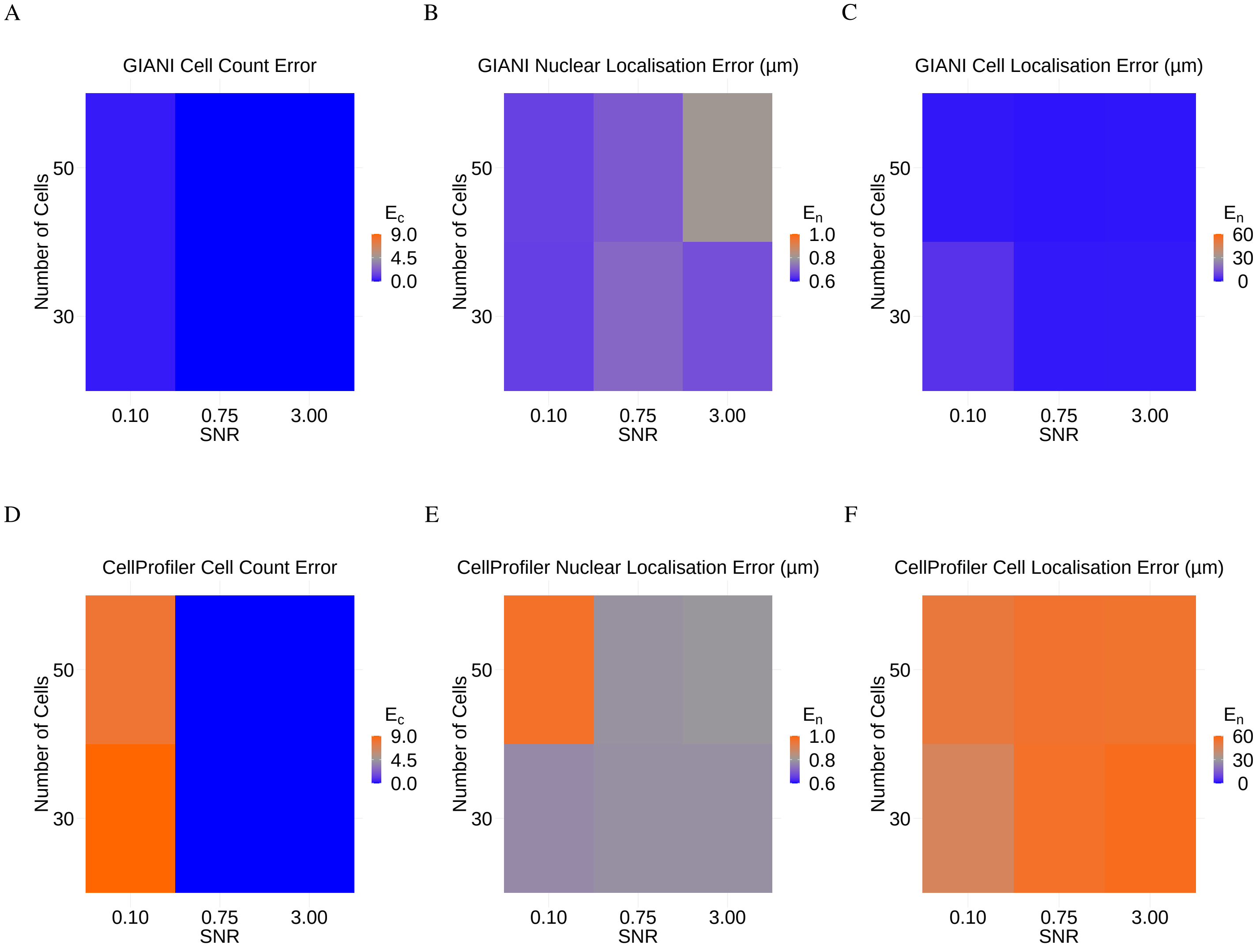

Fig. S2. Accuracy of simulated nuclei and cell detection and localisation by GIANI and CellProfiler compared. The data shown in A - C is a subset of that shown in Fig 3F - H. In D - F, each tile represents a single simulated embryo, which was reduced in size to 512 x 512 x 112 voxels prior to running the pipeline. A: Absolute errors in cell counts (E_c) produced by GIANI for

Journal of Cell Science • Supplementary information

simulated embryos with the indicated number of cells and signal-to-noise ratios (SNR). B: Absolute errors in nuclear centroid localisation (E_{nl}) produced by GIANI. C: Absolute errors in cell centroid localisation error (E_{cl}) produced by GIANI. D: Absolute errors in cell counts produced by CellProfiler. E: Absolute errors in nuclear centroid localisation produced by CellProfiler. F: Absolute errors in cell centroid localisation error produced by CellProfiler. The CellProfiler pipeline used and raw data are available to download from: https://dx.doi.org/10.5281/zenodo.5286507.

A

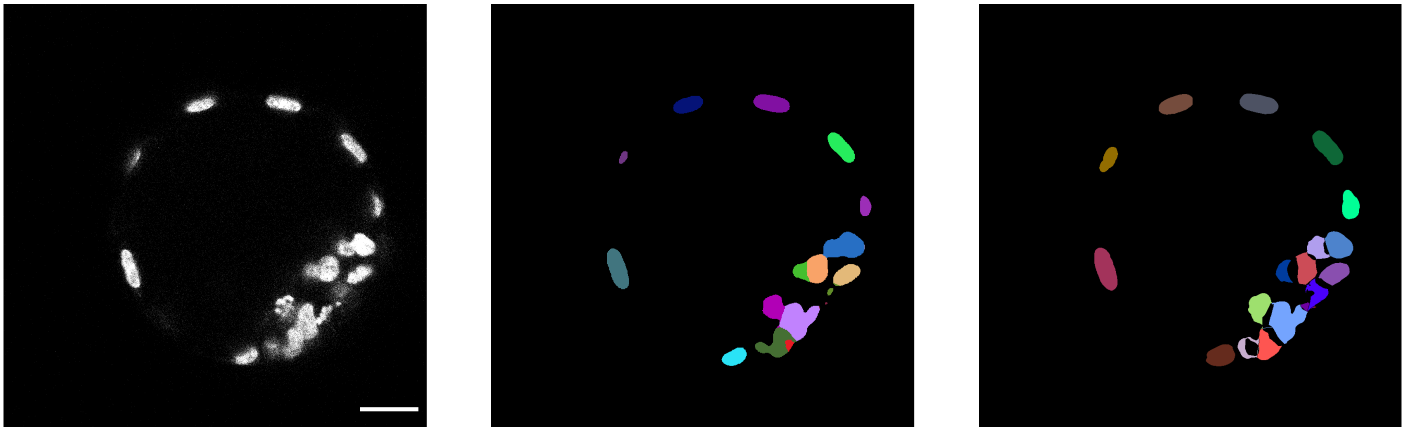

B

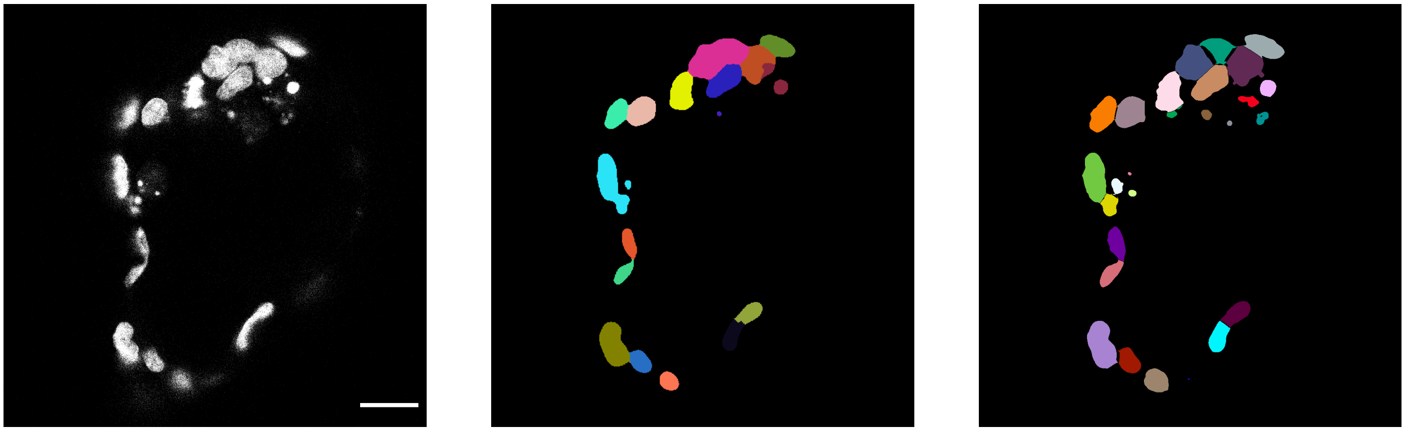

Raw Imaris GIANI

Fig. S3. Comparison of mouse blastocyst nuclear segmentations between GIANI and Imaris.. A single slice of (A) expanded and (B) hatching blastocysts are shown, together with the segmentations produced by Imaris and GIANI. Scale bars are all equivalent to 20 μm. The GIANI and Imaris settings used to produce this data, together with the raw image data, are available to download from https://dx.doi.org/10.5281/zenodo.5286670.

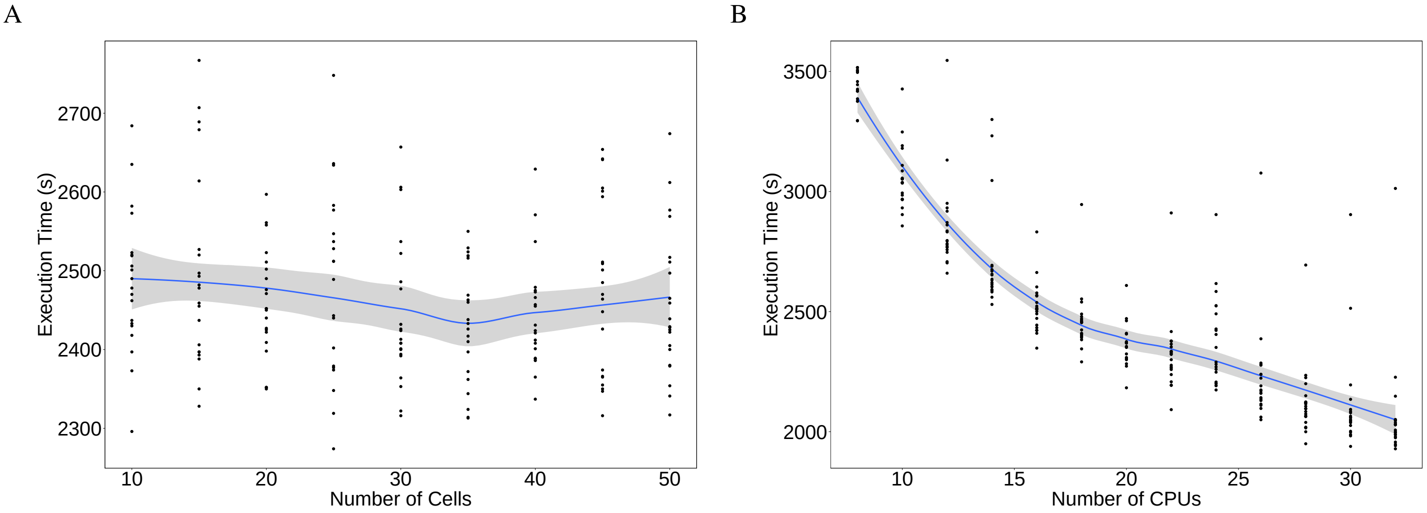

Fig. S4. Execution time of GIANI is independent of cell number, but decreases with increasing CPU availability. A: The length of time taken by GIANI to analyse simulated embryos versus the number of cells in the embryo. Each point represents the execution time for a single embryo - approximately 20 embryos were analysed for each cell number. The blue line represents a moving average calculated with LOESS smoothing. The grey bands represent the 95% confidence interval. B: The length of time taken by GIANI to analyse simulated embryos consisting of 30 cells versus the number of available CPUs. Each point represents the execution time for a single embryo - approximately 20 embryos were analysed for each CPU number. The blue line represents a moving average calculated with LOESS smoothing. The grey bands represent the 95% confidence interval.

Journal of Cell Science • Supplementary information

Table S1. Primary antibodies used in this study and the dilution at which they were used.

Antibody	Supplier	Catalogue Number	Dilution
YAP1	Abnova	H00010413-M01	1:50
GATA3	R&D	AF2605	1:200
E-CADHERIN	Life Technologies	131900	1:400

Journal of Cell Science • Supplementary information
