# Supplementary material for: GIANI – open-source software for automated analysis of 3D microscopy images
Source: J Cell Sci. 2022 May 17;135(10):jcs259511. doi: 10.1242/jcs.259511 (PMC9189431; doi:10.1242/jcs.259511)
Supplement: Reviewer comments [file joces-135-259511_review_history.pdf]

GIANI: open-source software for automated analysis of 3D microscopy images

David J. Barry, Claudia Gerri, Donald M. Bell, Rocco D'Antuono and Kathy K. Niakan
DOI: 10.1242/jcs.259511

Editor: Jennifer Lippincott-Schwartz

Review timeline

Original submission:	26 October 2021
Editorial decision:	21 December 2021
First revision received:	22 February 2022
Editorial decision:	4 April 2022
Second revision received:	13 April 2022
Accepted:	20 April 2022

Original submission

First decision letter

MS ID#: JOCES/2021/259511

MS TITLE: GIANI: open-source software for automated analysis of 3D microscopy images

AUTHORS: David J Barry, Claudia Gerri, Donald M Bell, Rocco D'Antuono, and Kathy K Niakan
ARTICLE TYPE: Tools and Resources

We have now reached a decision on the above manuscript.

To see the reviewers' reports and a copy of this decision letter, please go to: https://submit-jcs.biologists.org and click on the 'Manuscripts with Decisions' queue in the Author Area. (Corresponding author only has access to reviews.)

As you will see, the reviewers raise a number of substantial criticisms that prevent me from accepting the paper at this stage. They suggest, however, that a revised version might prove acceptable, if you can address their concerns. If you think that you can deal satisfactorily with the criticisms on revision, I would be pleased to see a revised manuscript. We would then return it to the reviewers.

We are aware that you may be experiencing disruption to the normal running of your lab that makes experimental revisions challenging. If it would be helpful, we encourage you to contact us to discuss your revision in greater detail. Please send us a point-by-point response indicating where you are able to address concerns raised (either experimentally or by changes to the text) and where you will not be able to do so within the normal timeframe of a revision. We will then provide further guidance. Please also note that we are happy to extend revision timeframes as necessary.

Please ensure that you clearly highlight all changes made in the revised manuscript. Please avoid using 'Tracked changes' in Word files as these are lost in PDF conversion.

I should be grateful if you would also provide a point-by-point response detailing how you have dealt with the points raised by the reviewers in the 'Response to Reviewers' box. Please attend to

© 2022. Published by The Company of Biologists under the terms of the Creative Commons Attribution License (https://creativecommons.org/licenses/by/4.0/).

all of the reviewers' comments. If you do not agree with any of their criticisms or suggestions please explain clearly why this is so.

Reviewer 1

Advance summary and potential significance to field

Creating a tool in FIJI, often the first tool a novice microscopist/image analyst will interact with, is therefore a nice way to get the tool into the hands of inexperienced users.

Comments for the author

The authors are correct that segmentation is important, yet often confusing for many inexperienced users. Unfortunately, the tool works only a finite set of conditions and is not terribly thoroughly documented (especially to the degree a novice user might need). I am not therefore convinced it makes a novice users' life any easier than, say, an example CellProfiler pipeline (the tool the authors pattern their approach on), Icy workflow, etc.
To begin with, users must enable multiple non-standard update sites in FIJI to access the tool- not challenging if you're an experienced FIJI user, and the authors lay out nicely on their GitHub wiki which update sites are needed, but this isn't an easier process than downloading a separate software tool. The tool, in my exploration, only works when all channels and Z planes are part of a single file- far from the only way microscopes export data, and the authors do not acknowledge this anywhere in their documentation nor provide guidance to their users on ways to get their data into compliance if it isn't already. Documentation within the tool is sparse at best- for example, their blob detector settings use the word "threshold" outside of its most common meaning in fluorescence analysis, and without going to the wiki side by side with the tool there is really no way to know this, and no simplified explanations for beginner users are available. Likewise with the smoothing settings- upon reading the manual, it is clear why the user may want to do some smoothing, and to what degree, but NONE of that is present within the tool itself. Users can only demo their settings on a single image, making it hard to know if a given set of settings will work on say, a positive and negative control image without running a whole data set (which, when single embryo images take 30-60 minutes, makes debugging very challenging). Ways to figure out reasonable starting values are provided for some steps of the manual, but not all. These design choices speak to me of a tool designed by a user who understands well EXACTLY what they need, and performing a tool that does it quite nicely, but without any extra functionality outside of that.
The authors have done some nice work here to show the tool can work nicely when calibrated correctly (and under what circumstances it performs best), and that it can detect known biological differences between samples; I have no doubt of its utility to the authors' own research, and that it could be useful to a user who is advanced enough to understand some segmentation theory but not comfortable with scripting on their own. The wiki is nicely put together in a step-by-step manner which incorporating into the tool could only help. But given that its approach is not novel, not is it designed in such a way to provide GREATER user interaction/ease of use than other existing tools, I am forced to conclude I am not sure where it would find a home amongst users in the broader bioimaging community.
The authors should also provide their entire simulated data set, not just a link to the code to generate it.

Reviewer 2

Advance summary and potential significance to field

The authors present GIANI, a user-friendly image analysis workflow design tool that comes in the form of a wizard. I'm sure the community will appreciate these efforts as guidance in building powerful image analysis workflows is key in an ecosystem with an overwhelming number of algorithms and tools available. GIANI is open source and freely available to the end-users community and I'm sure the community will appreciate it.

© 2022. Published by The Company of Biologists under the terms of the Creative Commons Attribution License (https://creativecommons.org/licenses/by/4.0/).

I'm wondering if GIANI is extensible. I'm sure the authors are aware that modern algorithms such as StarDist (DOI: 10.1109/WACV45572.2020.9093435) and CellPose (https://doi.org/10.1038/s41592-020-01018-x) are outperforming seeded-watershed approaches for cell-segmentation in many projects. Thus, an external developer might want to extend a GIANI workflow using those deep-learning based segmentation algorithms. If GIANI was easily extensible, GIANI could become a major player in user-friendly access to wizard-style workflow design.

Comments for the author

I think the manuscript could benefit from a screenshot of the user interface so that readers get an impression of how working with GIANI feels like. The first or second picture on https://github.com/djpbarry/Giani could be a nice example.
Page 3 / line 22. I'm not sure what "challenging to [..] automate for the uninitiated (FIJI ...)" means. From my perspective, the ImageJ macro recorder together with tools such as MorphoLibJ (https://doi.org/10.1093/bioinformatics/btw413) and the 3D ImageJ Suite (http://dx.doi.org/10.1093/bioinformatics/btt276) provide excellent opportunities for processing, analysis and automation for 3D microscopy data. Maybe this sentence just needs some rephrasing to make clear what benefit GIANI brings to users who are used to macro recording for 3D image data. Later in the text (page 9) it is clearer to read that GIANI aims to bring some CellProfiler-handiness to Fiji. One could argue that in that way the benefits from both platforms are combined (which is great).
Approximately on page 12 starts a virtual "Results" section even though the headline is missing. In the same section the authors state "extremely low" number of false positives and false negatives and increasing cell count errors. I would be happy if this could be more specific by providing concrete numbers, p-values etc in the main text. I would kindly ask the authors to seed some quantitative measurements of errors in all the sections on pages 12-14. Otherwise the reader has a hard time validating the authors' claims such as "performance of GIANI is comparable to Imaris" (page 13 / line 227).
There are text sections (e.g. Page 14 / lines 240-255) which read like a discussion of the results on the page before. I suggest to reorganize the manuscript in a "Results" and a "Discussion" section to better comply with community standards.

Minor comments:
Page 4 / line 44: I think it should be "truth" and not "truths".
Page 4 / line 50: "GIANI can be used to quantify 3D images..." I think it makes sense to clarify what it can quantify. I assume it's not "images". If this is to detailed here, I'd suggest to write "... to analyze 3D images" instead.
Page 5 / line 66: Please write "Euclidean" and not "euclidean" as this method is called after a person.
Page 8 / line 131, consider citing RStudio as the RStudio community suggests: https://support.rstudio.com/hc/en-us/articles/206212048-Citing-RStudio Page 8 / line 132: "known" instead of "know"
Page 8 / line 132, consider citing the Mann-Whitney-U test https://en.wikipedia.org/wiki/Mann%E2%80%93Whitney_U_test#cite_note-mannwhitney1947-1 Page 8 / line 116 + Page 9 / line 137: There are two independent sections about software implementation details. Consider merging both.
Page 9 / line 145 (figure caption): Is it possible that the "centroids from B" are in fact local maxima from a distance map or LoG image? I'm just wondering because I think the position of local maxima in such an image are not necessarily the centroids. Maybe I just have issues understanding Figure 1B (page 31). Some text next to the arrows what operation they correspond to might help.
Page 10 / line: The nuclei center position estimation is now more clear to me. I'm not sure if "centroid" is the right term. Consider rephrasing to something like "center of mass", "nuclei centers" or "nuclei positions". I'd say the centroid of a nucleus is mathematically differently defined (e.g. see scikit-image documentation https://scikit-image.org/docs/dev/api/skimage.measure.html#moments).
I also agree that the presented method is a good approximation of the centroid though.
Page 11 / line 184: Consider rephrasing "downsize" to "downsample".
Page 13 / line 217: "extremely low", I would either provide numbers before judging some quantitative measurement to be "extremely low" or just remove the "extreme" here.

© 2022. Published by The Company of Biologists under the terms of the Creative Commons Attribution License (https://creativecommons.org/licenses/by/4.0/).

Page 13 / line 230: "CellProfiler [...] errors far larger than this produced by GIANI." If the authors provide such statements, I suggest to provide concrete numbers for supporting these claims in the text.
Page 13 / line 236 - 239: Here is a short section about extensibility and future work in the middle of some results section. I suggest moving this sentence further down.
Page 16 / line 290 "although it fell slightly short of being statistically significant". I'm not sure if I can follow this statement. Consider rephrasing or removing.
Page 18 / line 328: The word "Finally" appears inappropriate here as we are in the middle of the text.
Page 18 / line 330: Consider writing "resulting" instead of "resultant".
Page 18 / line 330: I suggest to rephrase "less than perfect" and "at least comparable" to be more precise.
Page 18 / line 341 and following sections: We are now switching back to a methods section. I suggest reordering the manuscript to a structure like Methods ->
Results -> Discussion.
Page 19 / line 355: How are "success rates" defined? I'm not sure if this quantitative measurement was explained earlier.
Page 19 / line 362: "Give the longest dimension..." I like the thoughts here but think this belongs in a discussion section. Also, what would the authors conclude from this statement - an upper bound of an acceptable position error?
Page 20 / line 370: Please cite the Cell Tracking Challenge properly
(https://doi.org/10.1038/nmeth.4473)
Page 35 / figure 5: It appears that the Imaris dataset had a different pixel/voxel size compared to the GIANI result. Is that the case?
Sincerely,
Robert Haase robert.haase@tu-dresden.de

First revision

Author response to reviewers' comments

Reviewer 1
Advance Summary and Potential Significance to Field...

Creating a tool in FIJI, often the first tool a novice microscopist/image analyst will interact with, is therefore a nice way to get the tool into the hands of inexperienced users.

Comments for the Author...

The authors are correct that segmentation is important, yet often confusing for many inexperienced users. Unfortunately, the tool works only a finite set of conditions and is not terribly thoroughly documented (especially to the degree a novice user might need). I am not therefore convinced it makes a novice users' life any easier than, say, an example CellProfiler pipeline (the tool the authors pattern their approach on), Icy workflow, etc.

We appreciate the reviewer's concern with regard to accessibility and agree that ease of use, relative to existing tools such as CellProfiler, is paramount. However, as mentioned in the text (Line 16), CellProfiler does unfortunately not fully support the analysis of 3D datasets at this time. The lack of support for 3D data among existing software packages was the main motivation behind the development of GIANI. But we certainly do not want to claim it to be any easier to use than CellProfiler (for example), which, despite some limitations, we consider to be an excellent tool.

We have provided extensive documentation online (github.com/djpbarry/Giani/wiki), which includes step-by-step illustrated instructions on how to use GIANI. We also demonstrate, within our manuscript, that GIANI can be run on a variety of different sample types. For each of those examples, we have provided the parameter files produced by GIANI, for interested readers to

© 2022. Published by The Company of Biologists under the terms of the Creative Commons Attribution License (https://creativecommons.org/licenses/by/4.0/).

use as starting points in their own analyses.

To begin with, users must enable multiple non-standard update sites in FIJI to access the tool- not challenging if you're an experienced FIJI user, and the authors lay out nicely on their GitHub wiki which update sites are needed, but this isn't an easier process than downloading a separate software tool.

We chose this approach for distribution as it is considered “best practice” for the distribution of FIJI plugins (https://imagej.net/Distribution). While not ideal, it is preferable to the alternative, whereby users would have to manually download files themselves and ensure that they are placed in the correct directory. They would also have to ensure that they had the correct version of each file installed and would have to repeat this process every time GIANI (or one of its dependencies) was updated. On the other hand, update sites only need to be enabled once, after which the FIJI updater will automatically ensure that the user always has the most up-to-date version of GIANI installed - no further action is required from the user.

To make the whole process as straightforward as possible, we have updated the installation instructions on the GIANI wiki: github.com/djpbarry/Giani/wiki/Installation

The tool, in my exploration, only works when all channels and Z planes are part of a single file - far from the only way microscopes export data, and the authors do not acknowledge this anywhere in their documentation nor provide guidance to their users on ways to get their data into compliance if it isn't already.

GIANI uses Bio-Formats to load data and can therefore load any datasets recognised by Bio-Formats, regardless of how many files the data has been saved in (https://docs.openmicroscopy.org/bio- formats/6.6.0/supported-formats.html; Lines 66-73). To illustrate this point, we have updated the test data distributed with GIANI (github.com/djpbarry/Giani/wiki/Test-Data) to include the same image data in two different forms: in one case, all the data is contained in a single file, while in the other, each image channel is stored in a separate file. GIANI produces the same result, regardless of which dataset is used as input.

Documentation within the tool is sparse at best- for example, their blob detector settings use the word "threshold" outside of its most common meaning in fluorescence analysis, and without going to the wiki side by side with the tool there is really no way to know this, and no simplified explanations for beginner users are available. Likewise with the smoothing settings- upon reading the manual, it is clear why the user may want to do some smoothing, and to what degree, but NONE of that is present within the tool itself.

We acknowledge that the interface is somewhat rudimentary and this is something we intend to address in a future release. We found that incorporating the documentation directly into the GUI itself resulted in an extremely cluttered display. Indeed, most leading software packages in the field, such as, for example, ImageJ/FIJI (imagej.net/learn/), QuPath (qupath.readthedocs.io) and ilastik (www.ilastik.org/documentation), or other FIJI plugins, such as MorphoLibJ (imagej.net/plugins/morpholibj) and TrackMate (imagej.net/plugins/trackmate/), present their documentation online - we followed the same convention.
However, we have amended the labels in the GUI to make it clearer what each parameter controls. Hovering the mouse cursor over any text field or drop-down dialog will result in a tip being displayed, offering a brief explanation of what that parameter does. If further explanation is needed, there is a help button on each panel of the GUI that opens the relevant page in the online documentation describing what the parameters on the panel control.

Users can only demo their settings on a single image, making it hard to know if a given set of settings will work on say, a positive and negative control image without running a whole data set (which, when single embryo images take 30-60 minutes, makes debugging very challenging).

© 2022. Published by The Company of Biologists under the terms of the Creative Commons Attribution License (https://creativecommons.org/licenses/by/4.0/).

GIANI can be demoed on as many or as few images as the user wishes. The user can move forwards and backwards through the wizard-based GUI and they can also change the preview dataset used to make comparisons easier to visualise.

Furthermore, there is no need to run an entire analysis to see the effects of settings - the GUI is designed in a modular fashion such that each step of the analysis can be individually previewed, so settings can be refined in a step-by-step manner.

It should also be pointed out that the 30 - 60 minute analysis time referenced above refers to the total time needed to analyse one of our simulated datasets, each of which is approximately 2.1 GB in size. The experimental datasets we analysed were approximately 10 times smaller - for these datasets, the time taken to execute each step in the analysis pipeline ranges from a few seconds (for filtering, for example) to 1 or 2 minutes. But as is the case with any analysis software, these execution times will vary depending on the parameters used.

Ways to figure out reasonable starting values are provided for some steps of the manual, but not all.

In the documentation, we have endeavoured to explain in detail the purpose of each parameter in the interface, such that users can make informed decisions on appropriate values to use. But we are reluctant to suggest specific (or default) parameter values, as, given the potentially very broad utility of GIANI, it is very likely that some users will find the suggested values completely inappropriate for their data. That said, we have included in the supplemental data parameter files for all the analyses presented in our manuscript, so readers can use these as starting points for their own work.

These design choices speak to me of a tool designed by a user who understands well EXACTLY what they need, and performing a tool that does it quite nicely, but without any extra functionality outside of that.

While it is true to say GIANI was designed with a specific purpose in mind (routine analysis of nuclei- based images), that purpose is very broad and covers a wide range of biological samples. We have demonstrated the utility of GIANI on a variety of different datasets to illustrate its general applicability and believe that there is a very wide variety of sample types for which it is suitable.

The authors have done some nice work here to show the tool can work nicely when calibrated correctly (and under what circumstances it performs best), and that it can detect known biological differences between samples; I have no doubt of its utility to the authors' own research, and that it could be useful to a user who is advanced enough to understand some segmentation theory but not comfortable with scripting on their own. The wiki is nicely put together in a step-by-step manner, which incorporating into the tool could only help.

We thank the reviewer for their positive comments.

But given that its approach is not novel, not is it designed in such a way to provide GREATER user interaction/ease of use than other existing tools, I am forced to conclude I am not sure where it would find a home amongst users in the broader bioimaging community.

As outlined in our introduction (Lines 15-26), there are few, if any, freely-available open source tools for the analysis of 3D cell-based images. While such functionality does exist within FIJI, it is distributed across multiple independent plugins (requiring interaction with multiple different interfaces), making routine analysis of such images challenging for novices. Our goal in developing GIANI was to take all of that existing functionality in FIJI, assemble it into a reusable pipeline and make everything accessible via a single interface. For this reason, we feel that the approach we developed would be of interest to a diverse bioimaging community.

The authors should also provide their entire simulated data set, not just a link to the code to generate it.

© 2022. Published by The Company of Biologists under the terms of the Creative Commons Attribution License (https://creativecommons.org/licenses/by/4.0/).

All data is now publicly available on either the BioImage Archive or Zenodo (Lines 389-392).

Reviewer 2
Advance Summary and Potential Significance to Field...

The authors present GIANI, a user-friendly image analysis workflow design tool that comes in the form of a wizard. I'm sure the community will appreciate these efforts as guidance in building powerful image analysis workflows is key in an ecosystem with an overwhelming number of algorithms and tools available. GIANI is open source and freely available to the end-users community and I'm sure the community will appreciate it.

We thank the reviewer for their positive comments.

I'm wondering if GIANI is extensible. I'm sure the authors are aware that modern algorithms such as StarDist (DOI: 10.1109/WACV45572.2020.9093435) and CellPose (https://doi.org/10.1038/s41592- 020-01018-x) are outperforming seeded-watershed approaches for cell-segmentation in many projects. Thus, an external developer might want to extend a GIANI workflow using those deep- learning based segmentation algorithms. If GIANI was easily extensible, GIANI could become a major player in user-friendly access to wizard-style workflow design.

This is an excellent point and yes, GIANI is easily extensible for Java developers. We have added a template to the online documentation illustrating how developers can modify the default pipeline that GIANI uses and also write their own modules for incorporation into the pipeline (github.com/djpbarry/Giani/wiki/Extending-GIANI). We have also added a page on accessing GIANI using the ImageJ Macro Language (github.com/djpbarry/Giani/wiki/Scripting-with-GIANI), which does not offer the same level of flexibility, but may still be useful for some intermediate FIJI users.

Comments for the Author...

I think the manuscript could benefit from a screenshot of the user interface so that readers get an impression of how working with GIANI feels like. The first or second picture on https://github.com/djpbarry/Giani could be a nice example.
We thank the reviewer for this suggestion - we have now included a screenshot of the interface in Figure 1.

Page 3 / line 22. I'm not sure what "challenging to [..] automate for the uninitiated (FIJI ...)" means. From my perspective, the ImageJ macro recorder together with tools such as MorphoLibJ (https://doi.org/10.1093/bioinformatics/btw413) and the 3D ImageJ Suite (http://dx.doi.org/10.1093/bioinformatics/btt276) provide excellent opportunities for processing, analysis and automation for 3D microscopy data. Maybe this sentence just needs some rephrasing to make clear what benefit GIANI brings to users who are used to macro recording for 3D image data. Later in the text (page 9) it is clearer to read that GIANI aims to bring some CellProfiler-handiness to Fiji. One could argue that in that way the benefits from both platforms are combined (which is great).

We agree that this was poorly worded and have now modified the text to clarify (Lines 17-19). Our intended meaning was that while the functionality certainly exists within FIJI to perform analysis of 3D datasets (and much of this functionality is harnessed by GIANI), this would potentially require a complex series of commands using multiple different plugins (and associated interfaces). GIANI aims to gather all of this functionality into a single interface and assemble it into a generally-applicable pipeline. Furthermore, automating such an analysis across multiple datasets would be challenging, if not impossible, for a novice user with no knowledge of scripting, whereas GIANI facilitates automation - once analysis parameters are set, the same analysis can be run on multiple datasets.

Approximately on page 12 starts a virtual "Results" section even though the headline is missing. In the same section the authors state "extremely low" number of false positives and false negatives and increasing cell count errors. I would be happy if this could be more specific by

© 2022. Published by The Company of Biologists under the terms of the Creative Commons Attribution License (https://creativecommons.org/licenses/by/4.0/).

providing concrete numbers, p-values etc in the main text. I would kindly ask the authors to seed some quantitative measurements of errors in all the sections on pages 12-14. Otherwise the reader has a hard time validating the authors' claims such as "performance of GIANI is comparable to Imaris" (page 13 / line 227).

We agree with the reviewer - while quantitative data was presented in the figures referred to in this section, the text summarising the figures was very qualitative in nature. As such, we have revised this section, providing more concrete numbers concerning the accuracy of GIANI's analysis of simulated data and how it compared to Imaris (Lines 130-144). We have also added additional panels to Figure 2 and Supplemental Figure 1 to aid in the interpretation of the data.

There are text sections (e.g. Page 14 / lines 240-255) which read like a discussion of the results on the page before. I suggest to reorganize the manuscript in a "Results" and a "Discussion" section to better comply with community standards.

We have reformatted the manuscript as suggested and split the Results and Discussion section into two separate sections.

Minor comments:

Page 4 / line 44: I think it should be "truth" and not "truths". Page 4 / line 50: "GIANI can be used to quantify 3D images..." I think it makes sense to clarify what it can quantify. I assume it's not "images". If this is to detailed here, I'd suggest to write "... to analyze 3D images" instead.

We agree with the reviewer and have modified the text accordingly (Line 41).

Page 5 / line 66: Please write "Euclidean" and not "euclidean" as this method is called after a person.

We have modified the text accordingly (Line 324).

Page 8 / line 131, consider citing RStudio as the RStudio community suggests: https://support.rstudio.com/hc/en-us/articles/206212048-Citing-RStudio

We thank the reviewer for this suggestion - RStudio is now appropriately cited (Line 376).

Page 8 / line 132: "known" instead of "know"

We have modified the text accordingly (Line 377).

Page 8 / line 132, consider citing the Mann-Whitney-U test, https://en.wikipedia.org/wiki/Mann\%E2\%80\%93Whitney_U_test#cite_notemannwhitney1947-1

We thank the reviewer for this suggestion - the Mann-Whitney test is now appropriately cited (Line 377).

Page 8 / line 116 + Page 9 / line 137: There are two independent sections about software implementation details. Consider merging both.
We have now combined both sections under one heading (Lines 46-63).

Page 9 / line 145 (figure caption): Is it possible that the "centroids from B" are in fact local maxima from a distance map or LoG image? I'm just wondering because I think the position of local maxima in such an image are not necessarily the centroids. Maybe I just have issues understanding Figure 1B (page 31). Some text next to the arrows what operation they correspond to might help.

We thank the reviewer for drawing our attention to this. We have added annotations to what is now Figure 2B to clarify. We have also removed references to “centroid” in the figure legend and also the text (Lines 104-105), substituting the term “nuclear centres”.

© 2022. Published by The Company of Biologists under the terms of the Creative Commons Attribution License (https://creativecommons.org/licenses/by/4.0/).

Page 10 / line: The nuclei center position estimation is now more clear to me. I'm not sure if "centroid" is the right term. Consider rephrasing to something like "center of mass", "nuclei centers" or "nuclei positions". I'd say the centroid of a nucleus is mathematically differently defined (e.g. see scikit-image documentation https://scikit-image.org/docs/dev/api/skimage.measure.html#moments). I also agree that the presented method is a good approximation of the centroid though.

We thank the reviewer for drawing our attention to this. We have removed references to "centroid" (Lines 104-105), substituting the term "nuclear centres". Our intended meaning was that these blob detectors provide an approximate location of the nuclear centroid, rather than being mathematically equivalent to it.

Page 11 / line 184: Consider rephrasing "downsize" to "downsample".

We thank the reviewer for this suggestion, but we feel the term "downsize" is more appropriate here, as the image is interpolated when reduced in size, rather than being sampled at regular intervals.

Page 13 / line 217: "extremely low", I would either provide numbers before judging some quantitative measurement to be "extremely low" or just remove the "extreme" here.

We agree with the reviewer and have removed such qualitative statements from the text (Lines - 130-133).

Page 13 / line 230: "CellProfiler [...] errors far larger than this produced by GIANI." If the authors provide such statements, I suggest to provide concrete numbers for supporting these claims in the text.

We again agree with the reviewer and have removed such qualitative statements from the text (Lines -143-144).

Page 13 / line 236 - 239: Here is a short section about extensibility and future work in the middle of some results section. I suggest moving this sentence further down.
This text has now been moved to the Discussion section (Lines 257-267)

Page 16 / line 290 "although it fell slightly short of being statistically significant". I'm not sure if I can follow this statement. Consider rephrasing or removing.
We agree that this statement is confusing and have modified it accordingly (Lines 177-178).

Page 18 / line 328: The word "Finally" appears inappropriate here as we are in the middle of the text.
We have modified the text accordingly (Line 212).

Page 18 / line 330: Consider writing "resulting" instead of "resultant".

Page 18 / line 330: I suggest to rephrase "less than perfect" and "at least comparable" to be more precise.

We have now simplified this line in the text (Line 214-216).

Page 18 / line 341 and following sections: We are now switching back to a methods section. I suggest reordering the manuscript to a structure like Methods→Results→Discussion.

While there is some reference to methodology in this section, we feel it is more appropriately placed in the Results section as it is primarily concerned with presenting the results of an analysis.

Page 19 / line 355: How are "success rates" defined? I'm not sure if this quantitative measurement was explained earlier.

© 2022. Published by The Company of Biologists under the terms of the Creative Commons Attribution License (https://creativecommons.org/licenses/by/4.0/).

We agree that this term is ambiguous. We have either rephrased (Lines 224 \& 236) or clarified what is meant (Line 239).
Page 19 / line 362: "Give the longest dimension..." I like the thoughts here but think this belongs in a discussion section. Also, what would the authors conclude from this statement - an upper bound of an acceptable position error?

We thank the reviewer for this suggestion, but we feel this text is better-placed in the Results section as it rounds off the presentation of the data in Figure 5. However, we have added a concluding statement (Lines 244-245). In short, we believe this suggests an upper limit on nuclear density for reliable nuclear detection.

Page 20 / line 370: Please cite the Cell Tracking Challenge properly (https://doi.org/10.1038/nmeth.4473)

We thank the reviewer for this suggestion - the Cell Tracking Challenge is now appropriately cited (Lines 250-251).

Page 35 / figure 5: It appears that the Imaris dataset had a different pixel/voxel size compared to the GIANI result. Is that the case?

We thank the reviewer for drawing our attention to this. We can confirm that the voxel size of the analysed dataset was exactly the same in both cases. Unfortunately, without knowing exactly the internal workings of Imaris, we cannot explain the rather blocky/pixelated nature of the segmentation it produces (relative to that produced by GIANI) and there does not appear to be any parameter that can be modified in Imaris to change this. We can only speculate that perhaps Imaris down-samples the dataset prior to segmentation.

Second decision letter

MS ID#: JOCES/2021/259511

MS TITLE: GIANI: open-source software for automated analysis of 3D microscopy images

AUTHORS: David J Barry, Claudia Gerri, Donald M Bell, Rocco D'Antuono, and Kathy K Niakan
ARTICLE TYPE: Tools and Resources

We have now reached a decision on the above manuscript.

To see the reviewers' reports and a copy of this decision letter, please go to: https://submit-jcs.biologists.organd click on the 'Manuscripts with Decisions' queue in the Author Area. (Corresponding author only has access to reviews.)

As you will see, the reviewers gave favourable reports but raised some critical points that will require amendments to your manuscript. In particular, reviewer #1 has made some comments about usage of the program that I hope you can address, specifically the last 3 points in the 'notes' section of his/her review. I hope that you will be able to carry these out because I would like to be able to accept your paper.

Please ensure that you clearly highlight all changes made in the revised manuscript. Please avoid using 'Tracked changes' in Word files as these are lost in PDF conversion.

I should be grateful if you would also provide a point-by-point response detailing how you have dealt with the points raised by the reviewers in the 'Response to Reviewers' box. Please attend to all of the reviewers' comments. If you do not agree with any of their criticisms or suggestions please explain clearly why this is so.

© 2022. Published by The Company of Biologists under the terms of the Creative Commons Attribution License (https://creativecommons.org/licenses/by/4.0/).

Reviewer 1

Advance summary and potential significance to field

A tool to simplify 3D image analysis in Fiji in the case where the user has nuclei and cells, and optionally also spots.

Comments for the author

I thank the authors for addressing many of my and reviewer 2's comments.
I still believe that this tool is under-documented for beginners and I still doubt its broader utility; the use case as far as I can tell is still extremely specific (for example, can I use GIANI if I have nuclei and spots but no cells? It doesn't seem so but I'm honestly uncertain). The tool does not as of yet have any outside engagement in terms of GitHub issues, image.sc forum posts, etc, and I strongly suggest if the authors are interested in attracting and maintaining engagement that they address at least some of the concerns below - sitting down a naive user and watching them use the tool without any creator explanation can be a great way to learn where the pain points are. However, I would not block publication at this time.
This is also obviously not the authors' fault, but none of their data is actually accessible at BioImage Archive - either through the website, connecting via FTP or Aspera. They should follow up with the Archive to address this before publication.

Notes:
I understand philosophically why the authors don't want to suggest default values for parameters, but then in certain cases they need to do a better job of explaining what the parameters actually do - ie "Quality of Simple Nuclear Centroid Detections", how is one measuring quality? On a scale of 0-1, 1-100, etc?
It seems like probably what it actually is is a "minimum pixel value to be counted", but I can't say for sure.
All of the following are true in Giani 3.2.2 (which per the packaging site is the most up to date version available as of mid-March 2022
- The folder selector is awkward ; since all of the screenshots are in PC I'm not sure how well if at all this works in Mac. One cannot actually select files, only a folder, and if one navigates inside the folder where the files live, Giani actually doubles the folder name at the end of the path (ie /Users/dummy/Downloads/TestData becomes /Users/dummy/Downloads/TestData/TestData), leading to an "invalid file" error. You have to click on the last folder, but not enter it, in order for Giani to work. As far as I can tell, there is not a way to just have subsets of files loaded in Mac.
- The tool tip sort of works, but it took me a while to figure it out (you have to hover over the VALUES, not the actual parameter name), and as far as I can tell is not actually documented in the wiki (per a GH search) or in the paper (per an adobe search)
- The updated test data set containing both single files and multi-files is NOT available at the designated link; that file contains a single file (and results) only. I could not get Giani to read out either a folder I had containing 3 channels, nor the Supplemental File 8 data, to read as multi-channel, and there was no documentation on what to do. It's fine to not support some stuff! But then explicitly say that you don't, and/or tell users how to convert to a format that you do.
- In many places it is specified that distances are in microns; this is not necessarily true. It appears to be whatever UNITS the image is saved in - if I change the properties of the image to, say, nm, and give a value 10, it does not show me 10um circles, it shows me 10 nm circles. Likewise if I change the units to pixels or a random word. As far as I can tell, this is also not documented.
- The top hat filtering absolutely trashed my image, adding tons of artifacts without any apparent value. The authors should consider allowing this step to be optional, or at least provide some guidance as to when this works well (and when it does not)

Reviewer 2

Advance summary and potential significance to field

The authors present GIANI, a user-friendly image analysis workflow design tool that comes in the form of a wizard. I'm sure the community will appreciate these efforts as guidance in building

© 2022. Published by The Company of Biologists under the terms of the Creative Commons Attribution License (https://creativecommons.org/licenses/by/4.0/).

powerful image analysis workflows is key in an ecosystem with an overwhelming number of algorithms and tools available. GIANI is open source and freely available to the end-users community and I'm sure the community will appreciate it.

Comments for the author

I'm in general satisfied with the updated manuscript. All my questions were answered. Great work. Apologies for the delay in reviewing btw.
Just one minor suggestion:
Page 11, line 237 "success rate (percentage of nuclei detected)". With this definition, I suggest to use the established term "sensitivity" instead of "success rate".

Sincerely,
Robert Haase robert.haase@tu-dresden.de

Second revision

Author response to reviewers' comments

Reviewer 1
Advance Summary and Potential Significance to Field...

A tool to simplify 3D image analysis in Fiji in the case where the user has nuclei and cells, and optionally also spots.

Comments for the Author...

I thank the authors for addressing many of my and reviewer 2's comments.

I still believe that this tool is under-documented for beginners and I still doubt its broader utility; the use case as far as I can tell is still extremely specific (for example, can I use GIANI if I have nuclei and spots but no cells? It doesn't seem so but I'm honestly uncertain).

Yes, GIANI can be used in this manner, as per the following publication (it requires the specification of a “dummy” channel to represent the cells):
https://doi.org/10.1371/journal.ppat.1009484.

GIANI is somewhat agnostic about what constitutes “nuclei” and “cells” – at the most basic level, all that is required for GIANI to run is for the images to contain blobs of some sort (and the “nuclei” and “cells” can be the same blobs if necessary – in other words, a separate channel representing cells is not strictly required). However, we hesitate to outline this very general idea in the documentation for fear that it will cause confusion. Once again, we have attempted to make a tool that is as easy as possible for any cell biologist to understand with a very simple workflow: detect nuclei, detect cells, make measurements.
What we will do is add more use cases to the documentation as and when we encounter them. GIANI is currently being used by a number of groups in-house at the Crick, for example, and, in time, we will add the results of their efforts to the wiki (with links to any relevant publications).

The tool does not as of yet have any outside engagement in terms of GitHub issues, image.sc forum posts, etc…

It is true that, as far as we are aware, GIANI is not yet widely used in the community. However, there are mitigating circumstances. For example, there has been little opportunity to present our software at symposia and conferences over the last couple of years.

…and I strongly suggest if the authors are interested in attracting and maintaining engagement that they address at least some of the concerns below - sitting down a naive user and watching

© 2022. Published by The Company of Biologists under the terms of the Creative Commons Attribution License (https://creativecommons.org/licenses/by/4.0/).

them use the tool without any creator explanation can be a great way to learn where the pain points are. However, I would not block publication at this time.

This is also obviously not the authors' fault, but none of their data is actually accessible at BioImage Archive - either through the website, connecting via FTP, or Aspera. They should follow up with the Archive to address this before publication.

We thank the reviewer for bringing this to our attention. We have double-checked with BioImage Archive and can confirm that the data is accessible at the following links:

https://www.ebi.ac.uk/biostudies/BioImages/studies/S-BIAD328

https://www.ebi.ac.uk/biostudies/studies/S-BIAD334

It is true however that downloading such large datasets via a web browser is unlikely to be successful – we will consult with our colleagues at the BioImage Archive to find the best solution for provision of the data to interested parties.

Notes:

I understand philosophically why the authors don't want to suggest default values for parameters, but then in certain cases they need to do a better job of explaining what the parameters actually do - ie "Quality of Simple Nuclear Centroid Detections", how is one measuring quality? On a scale of 0-1, 1-100, etc?

It seems like probably what it actually is is a "minimum pixel value to be counted", but I can't say for sure.

We have updated instructions on the wiki to attempt to provide greater clarity. For example, for the above-mentioned parameter:
"Quality of Simple Nuclear Centroid Detections: effectively a noise threshold - blobs detected with a strength below this tolerance value will be removed. Increasing this value will result in fewer blobs detected. An appropriate value for this threshold depends somewhat on the intensity range in the input image, although they are not directly related. Begin with a value of between 0 and 1 (which will very likely result in artefacts being detected) and increase until only nuclei are detected."

https://github.com/djpbarry/Giani/wiki/Estimating-the-centres-of-nuclei

For more detail on what this particular parameter is actually controlling algorithmically, we refer the reviewer to Figure 2B.

Again, we are hesitant to include detailed algorithmic descriptions within the documentation, for the sake of brevity and clarity, and instead refer users to the manuscript should they require more detailed information about the inner workings of GIANI.

All of the following are true in Giani 3.2.2 (which per the packaging site is the most up to date version available as of mid-March 2022

- The folder selector is awkward ; since all of the screenshots are in PC I'm not sure how well if at all this works in Mac. One cannot actually select files, only a folder, and if one navigates inside the folder where the files live, Giani actually doubles the folder name at the end of the path (ie /Users/dummy/Downloads/TestData becomes /Users/dummy/Downloads/TestData/TestData), leading to an "invalid file" error. You have to click on the last folder, but not enter it, in order for Giani to work. As far as I can tell, there is not a way to just have subsets of files loaded in Mac.

GIANI has been extensively tested on Windows, Linux and Mac. While we appreciate that the appearance of the interface will differ to some extent from one operating system (OS) to another, providing separate screenshots for every OS would make the documentation rather

© 2022. Published by The Company of Biologists under the terms of the Creative Commons Attribution License (https://creativecommons.org/licenses/by/4.0/).

unwieldy, while providing little in the way of additional information. GIANI uses the OS directory selection dialogue, which will be slightly different depending on the OS. It is not possible to select individual files in any OS, only a directory, again to keep things as simple as possible. But, if an invalid directory is specified, it will be highlighted in red in the Input Directory text field (and can be manually corrected in the text field if necessary) – GIANI cannot be run until a valid input directory is specified.

- The tool tip sort of works, but it took me a while to figure it out (you have to hover over the VALUES, not the actual parameter name), and as far as I can tell is not actually documented in the wiki (per a GH search) or in the paper (per an adobe search)

We have added the relevant information to the wiki:
https://github.com/djpbarry/Giani/wiki/Getting- Help

- The updated test data set containing both single files and multi-files is NOT available at the designated link; that file contains a single file (and results) only. I could not get Giani to read out either a folder I had containing 3 channels, nor the Supplemental File 8 data, to read as multi-channel, and there was no documentation on what to do. It's fine to not support some stuff! But then explicitly say that you don't, and/or tell users how to convert to a format that you do.

With regard to the test data, we have now updated the zip file at https://github.com/djpbarry/Giani/blob/master/assets/Test_Data.zip - this was an error on our part, so we offer our apologies. With regard to the supplemental file 8 data, this was formatted specifically for CellProfiler. However, we take the reviewer's general point and have added some brief instructions to the wiki on what to do if data is not being read correctly:

https://github.com/djpbarry/Giani/wiki/Supported-File-Formats

- In many places it is specified that distances are in microns; this is not necessarily true. It appears to be whatever UNITS the image is saved in - if I change the properties of the image to, say, nm, and give a value 10, it does not show me 10um circles, it shows me 10 nm circles. Likewise if I change the units to pixels or a random word. As far as I can tell, this is also not documented.

We thank the reviewer for drawing our attention to this. This was an oversight which has now been corrected – the correct units of measure are now read from the image metadata (if present). We have modified the GUI slightly to remove the units of measure from parameter names and place them alongside the text fields for greater clarity.

- The top hat filtering absolutely trashed my image, adding tons of artifacts without any apparent value. The authors should consider allowing this step to be optional, or at least provide some guidance as to when this works well (and when it does not)

We appreciate this suggestion from the reviewer and have now added this functionality – there is now a toggle button to allow users to enable/disable top hat filtering.

Reviewer 2 Advance Summary and Potential Significance to Field...

The authors present GIANI, a user-friendly image analysis workflow design tool that comes in the form of a wizard. I'm sure the community will appreciate these efforts as guidance in building powerful image analysis workflows is key in an ecosystem with an overwhelming number of algorithms and tools available. GIANI is open source and freely available to the end-users community and I'm sure the community will appreciate it.

We thank the reviewer for their supportive comments.

© 2022. Published by The Company of Biologists under the terms of the Creative Commons Attribution License (https://creativecommons.org/licenses/by/4.0/).

Reviewer 2
Comments for the Author...

I'm in general satisfied with the updated manuscript. All my questions were answered. Great work. Apologies for the delay in reviewing btw.

Just one minor suggestion:

Page 11, line 237 "success rate (percentage of nuclei detected)". With this definition, I suggest to use the established term "sensitivity" instead of "success rate".

We thank the reviewer for this suggestion and have amended the text accordingly.

Third decision letter

MS ID#: JOCES/2021/259511

MS TITLE: GIANI: open-source software for automated analysis of 3D microscopy images

AUTHORS: David J Barry, Claudia Gerri, Donald M Bell, Rocco D'Antuono, and Kathy K Niakan
ARTICLE TYPE: Tools and Resources

I am happy to tell you that your manuscript has been accepted for publication in Journal of Cell Science, pending standard ethics checks.

© 2022. Published by The Company of Biologists under the terms of the Creative Commons Attribution License (https://creativecommons.org/licenses/by/4.0/).
